# Random plasma glucose levels and cause-specific mortality among Chinese adults without known diabetes: an 11-year prospective study of 450,000 people

**DOI:** 10.1136/bmjdrc-2021-002495

**Published:** 2021-11-02

**Authors:** Jane Vermunt, Fiona Bragg, Jim Halsey, Ling Yang, Yiping Chen, Yu Guo, Huaidong Du, Fanwen Meng, Pei Pei, Canqing Yu, Jun Lv, Junshi Chen, Liming Li, Sarah Lewington, Zhengming Chen, the China Kadoorie Biobank Collaborative Group

**Affiliations:** 1 Nuffield Department of Population Health, University of Oxford, Oxford, Oxfordshire, UK; 2 Medical Research Council Population Health Research Unit at the University of Oxford, Oxford, Oxfordshire, UK; 3 Fuwai Hospital Chinese Academy of Medical Sciences, National Center for Cardiovascular Diseases, Fuwei Hospital, Beijing, China; 4 NCDs Prevention and Control Department, Liuzhou Centre for Disease Control and Prevention, Guangxi, China; 5 Chinese Academy of Medical Sciences, Beijing, China; 6 Department of Epidemiology and Biostatistics, School of Public Health, Peking University, Beijing, China; 7 Peking University Center for Public Health and Epidemic Preparedness & Response, Peking University, Beijing, China; 8 China National Center for Food Safety Risk Assessment, Beijing, China; 9 UKM Medical Molecular Biology Institute (UMBI), Universiti Kebangsaan Malaysia, Kuala Lumpur, Malaysia

**Keywords:** blood glucose, mortality, epidemiology

## Abstract

**Introduction:**

We examined the associations between long-term usual random plasma glucose (RPG) levels and cause-specific mortality risks among adults without known diabetes in China.

**Research design and methods:**

The China Kadoorie Biobank recruited 512,891 adults (59% women) aged 30–79 from 10 regions of China during 2004–2008. At baseline survey, and subsequent resurveys of a random subset of survivors, participants were interviewed and measurements collected, including on-site RPG testing. Cause of death was ascertained via linkage to local mortality registries. Cox regression yielded adjusted HR for all-cause and cause-specific mortality associated with usual levels of RPG.

**Results:**

During median 11 years’ follow-up, 37,214 deaths occurred among 452,993 participants without prior diagnosed diabetes or other chronic diseases. There were positive log-linear relationships between RPG and all-cause, cardiovascular disease (CVD) (n=14,209) and chronic kidney disease (CKD) (n=432) mortality down to usual RPG levels of at least 5.1 mmol/L. At RPG <11.1 mmol/L, each 1.0 mmol/L higher usual RPG was associated with adjusted HRs of 1.14 (95% CI 1.12 to 1.16), 1.16 (1.12 to 1.19) and 1.44 (1.22 to 1.70) for all-cause, CVD and CKD mortality, respectively. Usual RPG was positively associated with chronic liver disease (n=547; 1.45 (1.26 to 1.66)) and cancer (n=12,680; 1.12 (1.09 to 1.16)) mortality, but with comparably lower risks at baseline RPG ≥11.1 mmol/L. These associations persisted after excluding participants who developed diabetes during follow-up.

**Conclusions:**

Among Chinese adults without diabetes, higher RPG levels were associated with higher mortality risks from several major diseases, with no evidence of apparent thresholds below the cut-points for diabetes diagnosis.

**Significance of this study**

**What is already known about this subject?**

- Diabetes is a known risk factor for mortality from various vascular and non-vascular diseases, but the relevance of plasma glucose levels below the diabetes threshold remains uncertain.

**What are the new findings?**

- Among adults without known diabetes, there were positive and log-linear associations between usual random plasma glucose (RPG) and mortality from cardiovascular disease, chronic kidney disease, chronic liver disease, cancer and all causes, extending down to at least a usual RPG of 5.1 mmol/L.
- Approximately 15% higher risks of cardiovascular disease, cancer and all-cause mortality, and approximately 45% higher risks of mortality due to chronic kidney and chronic liver disease, were observed per 1 mmol/L higher usual RPG.
- These associations persisted after excluding participants who developed diabetes during the 11-year follow-up period, with the exception of modest attenuation of the association with chronic kidney disease mortality.
- Absolute excess mortality associated with higher RPG levels was predominantly attributable to cardiovascular disease and cancer.

**How might these results change the focus of research or clinical practice?**

- Glycemia, beyond its association with diabetes, may provide useful information for prediction of mortality risks.
- If the observed associations are causal, population level reductions in glycemia could contribute to significant reductions in mortality.

## Introduction

Diabetes is a known and important risk factor for mortality due to a range of vascular and non-vascular diseases, including ischemic heart disease (IHD), stroke, chronic kidney disease (CKD) and infectious diseases.^1 2^ However, it remains uncertain whether this reflects associations specific to diabetes, or whether glycemia may still be a major risk factor for mortality even below the conventional clinical threshold for diagnosing diabetes.

Findings of previous studies investigating the associations of glycemia with mortality risks are mixed.^3–5^ While there is good evidence showing lower risk of mortality from cardiovascular diseases (CVDs) at lower plasma glucose levels below the diagnostic threshold for diabetes,^4 6–8^ the relationship with other specific causes of mortality is much less clear. Many studies examining these relationships have lacked adequate power to examine the shape of associations throughout the full glycemic range, and have instead studied broad glycemic categories.^9–11^ Moreover, the majority of existing evidence is derived from Western populations,^4 11^ and there has been no report to-date of the associations of random plasma glucose (RPG) with cause-specific mortality.

In recent decades, there has been a marked and rapid rise in the prevalence of diabetes and pre-diabetes in China,^12 13^ highlighting the importance of glycemia as a risk factor. Moreover, the burden of many major chronic diseases in China differs importantly from those in Western populations (eg, higher rates of mortality due to stroke, chronic liver disease and certain cancers, and comparably low rates of IHD mortality).^14^ As such, investigation of the associations of glycemia with cause-specific mortality in China would be expected to provide valuable new evidence on these associations, to inform disease prediction and prevention efforts in China and elsewhere.

Using data from the nationwide, prospective China Kadoorie Biobank (CKB) study of 0.5 million men and women, we examine the association between RPG levels and cause-specific mortality among Chinese adults without known diabetes.

## Research design and methods

### Study population

Details of the CKB design, methods, and participants have been described previously.^15 16^ Briefly, the study population consisted of permanent, non-disabled residents aged 35–74 years recruited from five rural and five urban areas of China. The 10 study areas were selected from China’s nationally representative Disease Surveillance Points^17^ to ensure diversity of disease prevalence and risk factors, while taking into consideration local capacity, study logistics, and disease and death registry data quality. All eligible residents from 100 to 150 rural villages or urban committees in each study area were invited to participate. The response rate was ~30%, and between 2004 and 2008, 512,726 people (including ~13,000 outside the target age range, extending the actual range to 30–79 years) were enrolled in the study.

### Data collection

Demographic and lifestyle information, and personal and family medical history were collected via trained interviewer-administered laptop-based questionnaires. A range of physical measurements were undertaken, including anthropometric measures, blood pressure and pulse rate, using calibrated instruments and standardized protocols. A 10 mL non-fasting (except participants from one area (Zhejiang) who were asked to fast) venous blood sample was collected, with time since last eating recorded. Plasma glucose levels were measured immediately on-site using the SureStep Plus meter (LifeScan, Milpitas, California, USA). Participants with RPG levels ≥7.8 and <11.1 mmol/L were invited to return the following day for a fasting plasma glucose test. Participants who reported pre-existing doctor-diagnosed diabetes were defined as having known diabetes. Screen-detected diabetes was defined as no known diabetes and RPG ≥11.1 mmol/L.

Resurveys, including randomly selected samples of approximately 5% of surviving participants, were conducted in 2008 and 2013–2014. As well as collecting the same data as in the baseline survey, enhancements (eg, ECG, bone density and cIMT measurements) and modification of certain procedures (eg, use of the On Call Advanced glucometer (ACON, San Diego, California, USA) for measurement of plasma glucose levels in the second resurvey) were included.

### Morbidity and mortality follow-up

The vital status of participants was obtained periodically from local registries and through annual review of official residential records, health insurance data and active confirmation through visits to local communities. Cause-specific mortality was ascertained primarily from death certificates. If a participant died and had not sought medical attention prior to death (<5% of deaths), cause of death was obtained via verbal autopsy.^18^ Information on non-fatal diseases was collected through linkage (via unique national identification) with disease registries (for stroke, IHD, cancer and diabetes) and national health insurance claim databases (covering >98% of study participants). Cause of death was coded using the 10th International Classification of Diseases (ICD10) by trained staff blinded to participant baseline information. All ICD10 classified causes of death were included in the present analyses (online supplemental table S1). By January 1, 2018 (the censoring date for these analyses), <1% of participants (n=4724) were lost to follow-up.

10.1136/bmjdrc-2021-002495.supp1 Supplementary data

### Statistical analyses

Participants with previously diagnosed (but not screen-detected) diabetes at baseline (n=16 162), missing values for RPG (n=8341, due to a supply issue with testing strips) or body mass index (BMI) (n=2), or implausible or outlying values for systolic blood pressure (SBP), diastolic blood pressure, BMI, waist or hip circumference, or height (n=1058) were excluded. Participants with a self-reported history of physician diagnosed IHD, chronic liver disease, CKD, stroke, transient ischemic attack, or cancer were also excluded (n=37,969), leaving 452,993 participants for inclusion in the present analyses.

Baseline RPG was categorized (<4.8, 4.8–5.2, 5.3–5.7, 5.8–6.4, 6.5–7.5, 7.6–11.0, ≥11.1 mmol/L) to ensure a similar number of events in each category, additionally incorporating the diagnostic threshold for diabetes.^19^ Prevalence and mean values of baseline characteristics were calculated across RPG categories, standardized to the age, sex and study area structure of the total study population.

HRs for the associations of baseline RPG with all-cause and cause-specific mortality were estimated using Cox proportional hazards models stratified by age-at-risk (5 year age groups), sex and study area (10 regions), and adjusted for education level (no formal school/primary school/middle school/high school/technical school or college/university), smoking (never/occasional/ex-regular/current regular), alcohol drinking (never regular/ex-regular/occasional or seasonal/monthly/reduced intake/weekly), physical activity (metabolic equivalent of task (MET) hours per day), BMI, waist and hip circumference, and SBP. Group-specific variances were used to estimate 95% CIs for each HR, allowing comparisons between any RPG categories and not only with the reference category.^20^ Assuming the existence of log-linear associations, baseline RPG was examined as a continuous variable after additionally excluding participants with screen-detected diabetes at baseline (to avoid treatment bias resulting from subsequent diagnosis of diabetes). Examination of HRs for the first five and subsequent years of follow-up showed no strong evidence of departure from the proportional hazard’s assumption.

Repeat RPG measures at subsequent resurveys were used to correct for regression dilution bias arising due to within-person variation in RPG levels. The regression dilution ratio (RDR=0.45) was estimated as the mean of the slopes of regression lines between baseline RPG and RPG at each resurvey (approximating to the midpoint of follow-up), adjusted for age, sex, study area and fasting time.^21 22^ Differences between the overall mean RPG and mean RPG in each category were multiplied by the RDR and the resulting differences added to the overall mean RPG to estimate the mean usual RPG level in each category. Log HRs per 1.0 mmol/L higher baseline RPG were multiplied by the reciprocal of the RDR to estimate HRs per 1.0 mmol/L higher usual (longer-term average) RPG. Adjusted HRs for all-cause, CVD and cancer mortality per 1.0 mmol/L higher usual RPG were compared across strata of other baseline variables and χ^2^ values for trend and heterogeneity calculated. Sensitivity analyses were completed excluding deaths recorded during the first 3 years of follow-up and, separately, participants who developed diabetes during follow-up, from Zhejiang province or with poor self-rated health at baseline. Additional analyses examined associations further adjusted for fasting time and for the frequency of consumption of staple foods, animal products, and fresh fruit and vegetables.

Mortality rates per 1000 person-years were estimated for mortality due to CVD, cancer, CKD, chronic liver disease and other medical causes by multiplying adjusted HRs with 95% CIs by a common factor to make the weighted average match the CKB mortality rate.

All analyses were conducted using SAS V.9.4. Figures were produced using R V.3.6.2.

## Results

Among the 452,993 participants without known diabetes or other major chronic diseases at baseline, the mean (SD) age was 51 (11) years, 59% were women and mean baseline RPG was 5.9 (1.9) mmol/L (table 1). Higher RPG was associated with older age, higher prevalence of poor self-reported health, and higher levels of adiposity and SBP. There were inverse associations of RPG with educational attainment, daily physical activity level and fasting time.

**Table 1:** Characteristics of study participants by level of baseline random plasma glucose

Characteristics*	Random plasma glucose (mmol/L)							Total
	<4.8	4.8–5.2	5.3–5.7	5.8–6.4	6.5–7.5	7.6–11.0	>11.0	
Participants, n	81,915	92,458	91,578	88,270	57,837	33,074	7861	452,993
Mean random plasma glucose (SD), mmol/L	4.3 (0.7)	5.0 (0.7)	5.5 (0.7)	6.1 (0.7)	6.9 (0.7)	8.5 (0.7)	16.0 (0.7)	5.9 (1.9)
Mean fasting time (SD), hours	5.2 (4.7)	6.2 (4.7)	5.7 (4.7)	4.5 (4.7)	3.2 (4.7)	2.7 (4.7)	3.2 (4.7)	4.9 (4.9)
Mean age (SD), years	49 (10)	50 (10)	51 (10)	52 (10)	54 (10)	55 (10)	56 (10)	51 (11)
Women, %	49	57	62	64	64	60	58	59
Poor self-reported health, %	8	8	8	9	10	10	11	9
Socioeconomic and lifestyle factors								
Urban resident, %	32	43	47	47	45	48	51	43
6+ years education, %	57	52	49	46	44	42	41	49
Ever regular smoking, %								
Men	76	75	74	74	74	74	75	75
Women	2	3	3	3	3	4	5	3
Ever regular drinking, %								
Men	38	42	43	43	42	43	45	42
Women	2	3	3	3	3	3	3	3
Mean physical activity (SD), MET-h/day	23 (14)	23 (14)	22 (14)	21 (14)	20 (14)	19 (14)	17 (14)	22 (14)
Medications at baseline, %								
Statin	2	2	2	2	2	2	2	2
Aspirin	8	7	7	8	9	9	8	8
Anti-hypertensive	48	43	44	45	48	52	49	46
Anthropometry and physical measurements, mean (SD)								
BMI, kg/m^2^	23 (3)	23 (3)	24 (3)	24 (3)	24 (3)	24 (3)	25 (3)	24 (3)
Waist circumference, cm	78 (9)	79 (9)	80 (9)	80 (9)	81 (9)	83 (9)	86 (9)	80 (10)
Hip circumference, cm	90 (7)	91 (7)	91 (7)	91 (7)	91 (7)	92 (7)	92 (7)	91 (7)
SBP, mm Hg	128 (21)	129 (21)	130 (21)	131 (21)	132 (21)	136 (21)	142 (21)	130 (21)

P values for tests for trend across RPG categories <0.001 for all characteristics.

*Standardized to age, sex and study area structure of the population.

BMI, body mass index; MET-h/day, metabolic equivalent of task hours per day; SBP, systolic blood pressure.

**Figure 1:**
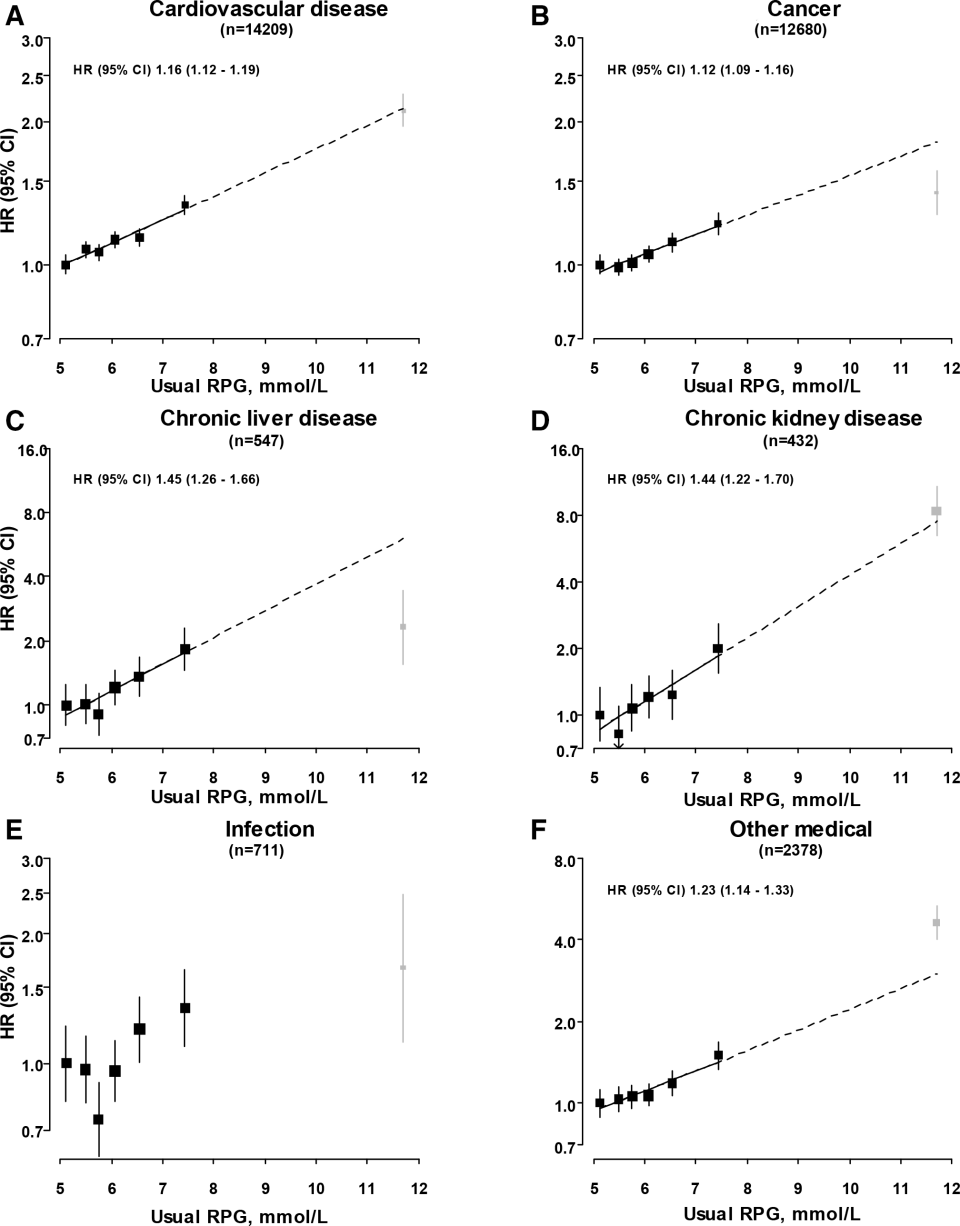
Associations of usual random plasma glucose with mortality from selected major diseases. HRs are presented stratified by age-at-risk, sex and study area, and adjusted for education, smoking, alcohol, physical activity, body mass index, waist and hip circumference, and systolic blood pressure. HRs are plotted against mean usual RPG level in each category. Squares represent the HR, with area inversely proportional to the variance of the log HR. Grey squares represent the HR among participants with screen-detected diabetes (baseline RPG ≥11.1 mmol/L). Vertical lines indicate the 95% CI. RPG, random plasma glucose.

**Figure 2:**
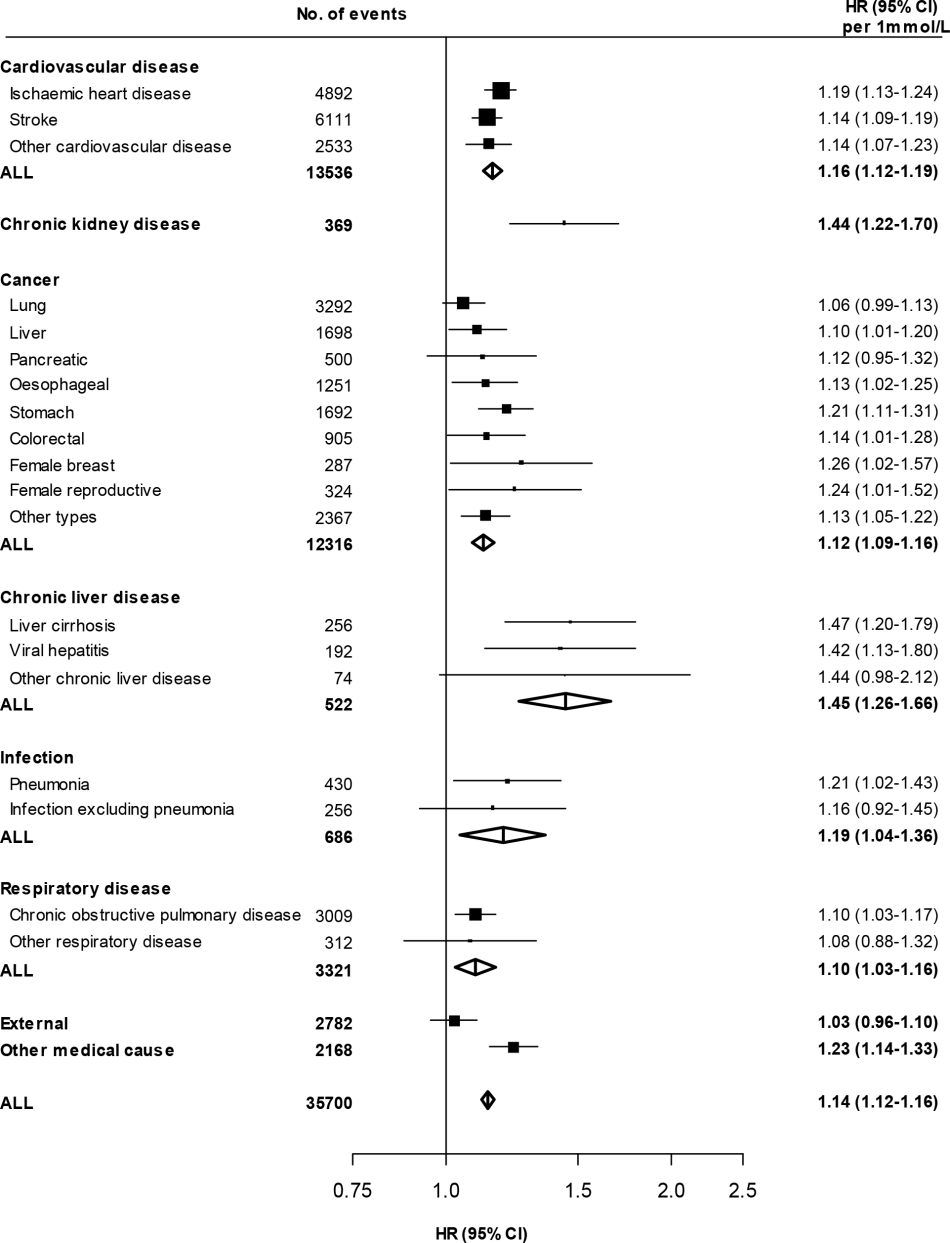
Associations of usual random plasma glucose with mortality from specific diseases and disease types. HRs are calculated per 1.0 mmol/L higher usual random plasma glucose. Results are stratified by age-at-risk, sex and study area and adjusted for education, smoking, alcohol, physical activity, body mass index, waist and hip circumference, and systolic blood pressure, except where the variable of interest. Participants with screen-detected diabetes at baseline are excluded. Squares represent the HR with area inversely proportional to the variance of the log HR. Horizontal lines represent the corresponding 95% CI. Open diamonds represent the overall HR for disease categories and their 95% CI. RPG, random plasma glucose.

During ~4.9 million person-years of follow-up (median (IQR) 11 (10–12)), 37,224 deaths were recorded, including 14,209 CVD, 12,680 cancer and 432 CKD deaths. A further 547 participants died due to chronic liver disease, 3422 from respiratory disease and 711 from infectious causes. Among participants included in the present analyses and without screen-detected diabetes at baseline, there were positive and log-linear associations of RPG levels with death from all-causes (online supplemental figure S1) and from mortality due to CVD, cancer, CKD and chronic liver disease, as well as other medical causes (figure 1). That is, a given absolute difference in usual RPG was associated with the same proportional difference in mortality risk throughout the RPG range studied. These associations extended down to a usual RPG level of at least 5.1 mmol/L, with no evidence of a threshold. There were similar strengths of association of RPG with all-cause (HR 1.14 (95% CI 1.12 to 1.16) per 1.0 mmol/L higher usual RPG) and overall CVD (1.16 (1.12 to 1.19)) mortality, as well as mortality due to major CVD types (IHD: 1.19 (1.13 to 1.24); stroke: 1.14 (1.09 to 1.19)) (figure 2). The slope of these associations remained unchanged at higher RPG levels, such that participants with screen-detected diabetes (baseline RPG ≥11.1 mmol/L) had HR of 1.93 (95% CI 1.84 to 2.04) and 2.11 (1.95 to 2.28) for all-cause and CVD mortality, respectively, when compared with participants with a usual RPG level <5.1 mmol/L. There was a somewhat weaker association of RPG with mortality due to cancer; each 1.0 mmol/L higher usual RPG was associated with 12% (HR 1.12 (95% CI 1.09 to 1.16)) higher risk (figure 1). The relatively small number of deaths due to individual cancer types limits inferences regarding their associations (online supplemental figure S2). However, there appeared to be a relatively strong positive and log-linear association of usual RPG with mortality from stomach cancer (1.21 (1.11 to 1.31)). The association with overall cancer mortality attenuated at the highest RPG levels (ie, among participants with screen-detected diabetes) (HR 1.42 (1.28 to 1.57)) and after exclusion of deaths occurring within the first 3 years of follow-up (1.09 (1.05 to 1.13) per 1.0 mmol/L higher usual RPG) (online supplemental table S2). With the exception of stronger associations of RPG with CVD mortality (online supplemental figure S3) at younger ages (p trend=0.01), there was no clear heterogeneity in the associations with all-cause (online supplemental figure S4), CVD or cancer mortality (online supplemental figure S5) across major population subgroups or study areas (online supplemental figure S6).

**Figure 3:**
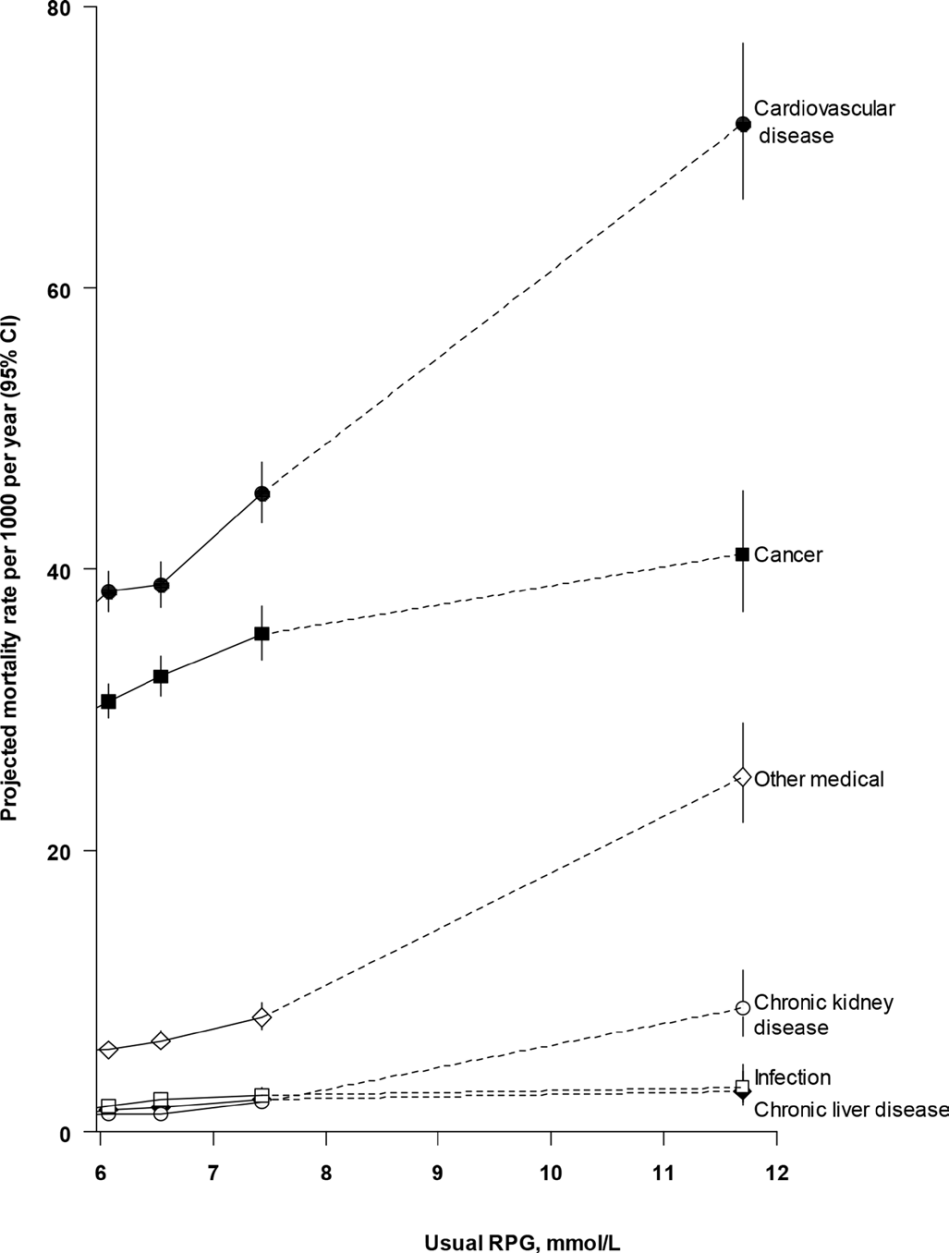
Projected absolute mortality rates from selected major diseases by levels of usual random plasma glucose. Mortality rates are adjusted for age, sex, region, education, smoking, alcohol, physical activity, body mass index, waist and hip circumference, and systolic blood pressure and are multiplied by a common factor (ie, floated) to make the weighted average match the China Kadoorie Biobank mortality rate. 95% CIs for floated rates reflect uncertainty in the log risk for each single rate. RPG, random plasma glucose.

There were strong positive log-linear associations of RPG with mortality due to CKD and chronic liver disease (figure 1) and largely consistent associations with mortality due to subtypes of chronic liver disease (online supplemental figure S7). Throughout the full glycemic range (extending up to usual RPG levels of at least 11.7 mmol/L), each 1.0 mmol/L higher usual RPG was associated with 44% higher risk of CKD mortality (HR 1.44 (95% CI 1.22 to 1.70)). This association persisted, but was moderately attenuated (1.34 (1.11 to 1.63)), after exclusion of participants who developed incident diabetes (n=21 548) during follow-up (online supplemental table S2). There was a similar association of RPG with death due to chronic liver disease (1.45 (1.26 to 1.66)). In contrast, however, the risk among participants with screen-detected diabetes was comparatively lower (2.31 (1.55 to 3.45)) than would be expected based on this association (figure 1). Exclusion of the first 3 years of follow-up attenuated the association of usual RPG with chronic liver disease mortality (n=406 cases; 1.32 (1.12 to 1.56)) (online supplemental table S2).

An apparent J-shaped association was observed between RPG levels and risk of mortality from infectious causes, with lowest risk at a usual RPG of ~5.8 mmol/L (figure 1). There were no clear associations of RPG levels with mortality from respiratory disease (online supplemental figure S8) or from external causes (HR 1.03 (95% CI 0.96 to 1.10) per 1.0 mmol/L higher usual RPG) (online supplemental figure S1).

The observed associations of RPG with major cause-specific mortality persisted after exclusion of participants from Zhejiang province (where participants were asked to fast prior to blood sampling), or with poor self-rated health at baseline (online supplemental table S2). Similarly, the associations remained largely unchanged after additional adjustment for fasting time and, separately, dietary variables.

Figure 3 shows absolute mortality rates due to six major disease types according to usual RPG levels. At all RPG levels, CVD accounted for the highest mortality rate, followed by cancer. CKD, chronic liver disease and infectious disease mortality rates were similar but significantly lower than those for CVD and cancer, and rates of mortality due to CKD increased at usual RPG levels ≥7.4 mmol/L.

## Conclusions

In this large prospective study of Chinese adults without known diabetes, each higher level of circulating RPG was associated with higher risk of mortality from various vascular and non-vascular diseases. There were positive and log-linear associations between usual RPG and mortality from CVD, CKD, chronic liver disease, cancer and all causes, extending down to at least usual RPG of 5.1 mmol/L, a level well below the diagnostic threshold for diabetes. Among participants without known or screen-detected diabetes at baseline, each 1.0 mmol/L higher usual RPG was associated with 12%–45% higher risks of mortality due to these causes. Moreover, these associations persisted after excluding participants who developed diabetes during follow-up. Mortality rates associated with RPG were highest for CVD and cancer throughout the glycemic range examined.

Previous large prospective studies have demonstrated positive associations between plasma glucose levels and risk of all-cause mortality, but there were differences in the shape of the associations.^9 23–25^ In contrast with the continuous positive association throughout the full glycemic range in our study, both the Korean Metabolic Risk Factor (KOMERIT) study, based on routinely collected health insurance data for ~12.5 million participants (~0.6 million deaths),^3^ and the predominantly Western population Emerging Risk Factors Collaboration (ERFC) individual participant data meta-analysis (including ~170,000 participants)^4^ observed J-shaped associations between baseline fasting plasma glucose levels and the risk of all-cause mortality. These studies both found approximately log-linear positive relationships at fasting plasma glucose levels above ~5.5 mmol/L (consistent with reported higher all-cause mortality risk among individuals with pre-diabetes^26^). The relatively higher mortality risks at the lowest fasting plasma glucose levels might reflect statistical artifact due to small numbers of deaths. Alternatively, it may reflect effects of residual reverse causality. Although both studies excluded participants with prior diabetes, the KOMERIT study did not exclude participants with other chronic diseases^3^ and the ERFC analyses excluded only those with prior CVD.^4^ On the other hand, the contrasting association when compared with CKB may be due to use of different glycemic measures.

The continuous positive relationship between RPG and CVD mortality in the present study is consistent with associations with incident major CVD in CKB,^6^ and with the association of fasting plasma glucose levels in the Asia Pacific Cohort Studies Collaboration individual participant data meta-analysis.^7^ This latter study, including 240,000 participants from 13 prospective studies, found a positive log-linear association with CVD mortality extending down to a usual fasting plasma glucose of at least 4.9 mmol/L.^7^ In contrast, analyses from the ERFC^4^ and the Korean Cancer Prevention Study (KCPS) (including ~1.2 million participants)^8^ showed J-shaped associations of fasting plasma glucose with CVD and atherosclerotic CVD mortality, respectively. Thresholds in these associations were observed at baseline fasting plasma glucose levels of 5.0–5.6 mmol/L, again possibly reflecting comparatively small numbers of deaths at the lowest fasting plasma glucose levels. Despite the apparent differences in the associations at the lower end of the glycemic range, in combination, these studies support an association of glycemia with CVD mortality risk below diabetes diagnostic thresholds, consistent with reported higher CVD risks associated with pre-diabetes,^26 27^ and with genetic studies suggesting a causal relationship of non-diabetic glycemia with atherosclerotic CVD.^28 29^

We demonstrated a continuous positive association between RPG and mortality due to CKD, which was stronger than that with CVD mortality, and which persisted after exclusion of participants who developed diabetes during follow-up. Although a positive association of glycemia with risk of CKD (largely renal microvascular disease) has been observed in people with diabetes,^30^ to our knowledge, this is the first study to observe an association with CKD mortality among individuals without known diabetes, independent of blood pressure and adiposity. With supportive evidence from a recent genetic study,^31^ this is of interest given the ongoing debate regarding the relevance of the diabetes diagnostic threshold to microvascular disease risk,^32 33^ and future investigation of the association with more refined CKD endpoints will be of interest. There was a strong association between usual RPG and mortality due to chronic liver disease, comparable to that with CKD mortality. However, attenuation of this association after exclusion of the first 3 years of follow-up suggests there may be residual reverse causality, possibly reflecting incomplete understanding of chronic liver disease natural history and etiological pathways linking non-alcoholic fatty liver disease with cardiometabolic diseases and their risk factors.

Consistent with our study findings, the prospective KCPS study reported a positive log-linear association of fasting plasma glucose with cancer mortality, based on ~26,000 cancer deaths.^5^ These contrast with the J-shaped association of fasting plasma glucose in the ERFC. However, this latter study included a much smaller number of cancer deaths (n=12,370).^4^ In combination with reported higher risks of overall and certain type-specific cancer mortality risks in diabetes,^2 34^ these findings lend support to a role for glycemia as a risk factor for cancer mortality. The comparatively lower cancer mortality risk among individuals with screen-detected diabetes in the present study, and the qualitatively similar findings in the ERFC analyses, might reflect reduced cancer risks reported with certain diabetes medications (eg, metformin).^35^ However, lack of data on the use of anti-hyperglycemic agents during follow-up precludes further investigation of this phenomenon. The current and previous studies have included too few cancer deaths to robustly investigate the associations of plasma glucose levels with mortality from specific cancer types,^4 5^ and findings to-date have been inconsistent.

This is the first prospective study to examine the associations of RPG with risks of cause-specific mortality. Although RPG is subject to greater intraindividual variation than other glycemic indicators (eg, fasting plasma glucose, HbA1c),^7 36^ additional adjustment for fasting time did not appreciably alter the shape or strength of the observed associations, consistent with the small proportion of variation in RPG levels accounted for by fasting time.^6^ Measurement of RPG levels is more practical, and, since for the majority of the time individuals do not exist in a fasting state, is arguably more relevant. In combination, these considerations highlight the value of RPG as a glycemic marker within the context of large-scale population-based studies.

Our study has several strengths, including the large study population, low loss to follow-up, and robust ascertainment of cause of death over a >10-year period. These enabled precise and reliable estimates of the independent associations of RPG with cause-specific mortality and detailed assessment of the shape of these associations. Furthermore, repeat RPG measurements allowed adjustment for regression dilution bias. Finally, the ability to consider separately participants with screen-detected diabetes at baseline, or to exclude those who developed incident diabetes during follow-up, provides confidence that the findings do not simply reflect the association of higher RPG levels with higher risk of future diabetes. However, the study has limitations. The small number of deaths for some endpoints resulted in imprecise estimates. Furthermore, it was not possible to adjust for other potentially relevant blood-based measures (eg, lipids), so the presented risk estimates may be subject to residual confounding. Finally, given the observational nature of the study, and the current lack of large-scale genetic data in CKB, causality cannot be inferred.

In summary, among participants without diabetes, there were positive log-linear associations of RPG levels with CVD, CKD, chronic liver disease, cancer and all-cause mortality, which extended throughout the full glycemic range examined and persisted after excluding those who developed diabetes during follow-up. This provides potential etiological insights and suggests glycemia, beyond its association with diabetes, may provide useful information for prediction of mortality risks. The causal nature of many of these associations is unclear. However, if established, despite the relatively weak associations of RPG with mortality risks, population level reductions in glycemia could contribute significantly to reducing mortality, and in particular mortality due to CVD and cancer.

## Data Availability

Data are available upon reasonable request. The China Kadoorie Biobank (CKB) is a global resource for the investigation of lifestyle, environmental, blood biochemical and genetic factors as determinants of common diseases. The CKB study group is committed to making the cohort data available to the scientific community in China, the UK and worldwide to advance knowledge about the causes, prevention and treatment of disease. For detailed information on what data is currently available to open access users and how to apply for it, visit: http://www.ckbiobank.org/site/Data+Access. Researchers who are interested in obtaining the raw data from the China Kadoorie Biobank study that underlines this paper should contact ckbaccess@ndph.ox.ac.uk. A research proposal will be requested to ensure that any analysis is performed by bona fide researchers and - where data is not currently available to open access researchers - is restricted to the topic covered in this paper.
