# Supplementary material for: Random plasma glucose levels and cause-specific mortality among Chinese adults without known diabetes: an 11-year prospective study of 450,000 people
Source: BMJ Open Diabetes Res Care. 2021 Nov 2;9(2):e002495. doi: 10.1136/bmjdrc-2021-002495 (PMC8565533; doi:10.1136/bmjdrc-2021-002495)
Supplement: Supplementary data [file bmjdrc-2021-002495supp001.pdf]

This page is intentionally left blank

Random plasma glucose levels and cause-specific mortality among Chinese adults without known diabetes: an 11-year prospective study of 450,000 people

Online-Only Supplemental Material, Table of Contents

	**Page**
China Kadoorie Biobank collaborative group and members	2
**Table S1.** ICD10 classified causes of death	3
**Table S2.** Sensitivity analyses of the associations of usual random plasma glucose with mortality from selected major diseases, applying additional adjustments and/or exclusions	4
**Table S3.** Associations of baseline random plasma glucose with mortality from selected major diseases	5
**Figure S1.** Associations of usual random plasma glucose with mortality from selected major diseases	6
**Figure S2.** Associations of usual random plasma glucose with cancer mortality	7
**Figure S3.** Associations of usual random plasma glucose with cardiovascular disease mortality	8
**Figure S4.** Associations of usual random plasma glucose with all-cause mortality in selected population subgroups	9
**Figure S5.** Associations of usual random plasma glucose with cancer mortality	10
**Figure S6.** Associations of usual random plasma glucose with all-cause, cardiovascular disease and cancer mortality by study area	11
**Figure S7.** Associations of usual random plasma glucose with liver disease mortality	12
**Figure S8.** Associations of usual random plasma glucose with respiratory disease mortality	13

Vermunt J, *et al. BMJ Open Diab Res Care* 2021; 9:e002495. doi: 10.1136/bmjdrc-2021-002495

China Kadoorie Biobank collaborative group and members

International Steering Committee: Junshi Chen, Zhengming Chen (PI), Robert Clarke, Rory Collins, Yu Guo, Liming Li (PI), Chen Wang, Jun Lv, Richard Peto, Robin Walters

International Co-ordinating Centre, Oxford: Daniel Avery, Fiona Bragg, Derrick Bennett, Ruth Boxall, Ka Hung Chan, Yumei Chang, Yiping Chen, Zhengming Chen, Johnathan Clarke; Robert Clarke, Huaidong Du, Zammy Fairhurst-Hunter, Hannah Fry, Wei Gan, Simon Gilbert, Alex Hacker, Parisa Hariri, Mike Hill, Michael Holmes, Pek Kei Im, Andri Iona, Maria Kakkoura, Christiana Kartsonaki, Rene Kerosi, Kuang Lin, Mohsen Mazidi, Iona Millwood, Qunhua Nie, Alfred Pozarickij, Paul Ryder, Sam Sansome, Dan Schmidt, Paul Sherliker, Rajani Sohoni, Becky Stevens, Iain Turnbull, Robin Walters, Lin Wang, Neil Wright, Ling Yang, Xiaoming Yang, Pang Yao

National Co-ordinating Centre, Beijing: Yu Guo, Xiao Han, Can Hou, Chun Li, Chao Liu, Jun Lv, Pei Pei, Canqing Yu

10 Regional Co-ordinating Centres:

Guangxi Provincial CDC: Naying Chen, Duo Liu, Zhenzhu Tang. Liuzhou CDC: Ningyu Chen, Qilian Jiang, Jian Lan, Mingqiang Li, Yun Liu, Fanwen Meng, Jinhuai Meng, Rong Pan, Yulu Qin, Ping Wang, Sisi Wang, Liuping Wei, Liyuan Zhou. **Gansu** Provincial CDC: Caixia Dong, Pengfei Ge, Xiaolan Ren. Maiji CDC: Zhongxiao Li, Enke Mao, Tao Wang, Hui Zhang, Xi Zhang. **Hainan** Provincial CDC: Jinyan Chen, Ximin Hu, Xiaohuan Wang. Meilan CDC: Zhendong Guo, Huimei Li, Yilei Li, Min Weng, Shukuan Wu. **Heilongjiang** Provincial CDC: Shichun Yan, Mingyuan Zou, Xue Zhou. Nangang CDC: Ziyan Guo, Quan Kang, Yanjie Li, Bo Yu, Qinai Xu. **Henan** Provincial CDC: Liang Chang, Lei Fan, Shixian Feng, Ding Zhang, Gang Zhou. Huixian CDC: Yulian Gao, Tianyou He, Pan He, Chen Hu, Huarong Sun, Xukui Zhang. **Hunan** Provincial CDC: Biyun Chen, Zhongxi Fu, Yuelong Huang, Huilin Liu, Qiaohua Xu, Li Yin. Liuyang CDC: Huajun Long, Xin Xu, Hao Zhang, Libo Zhang. **Jiangsu** Provincial CDC: Jian Su, Ran Tao, Ming Wu, Jie Yang, Jinyi Zhou, Yonglin Zhou. Suzhou CDC: Yihe Hu, Yujie Hua, Jianrong Jin Fang Liu, Jingchao Liu, Yan Lu, Liangcai Ma, Aiyu Tang, Jun Zhang. **Qingdao** Qingdao CDC: Liang Cheng, Ranran Du, Ruqin Gao, Feifei Li, Shanpeng Li, Yongmei Liu, Feng Ning, Zengchang Pang, Xiaohui Sun, Xiaocao Tian, Shaojie Wang, Yaoming Zhai, Hua Zhang, Licang CDC: Wei Hou, Silu Lv, Junzheng Wang. **Sichuan** Provincial CDC: Xiaofang Chen, Xianping Wu, Ningmei Zhang, Weiwei Zhou. Pengzhou CDC: Xiaofang Chen, Jianguo Li, Jiaqiu Liu, Guojin Luo, Qiang Sun, Xunfu Zhong. **Zhejiang** Provincial CDC: Weiwei Gong, Ruying Hu, Hao Wang, Meng Wan, Min Yu. Tongxiang CDC: Lingli Chen, Qijun Gu, Dongxia Pan, Chunmei Wang, Kaixu Xie, Xiaoyi Zhang

Table S1. ICD10 classified causes of death

Category	ICD-10 codes
All-cause mortality	Any ICD-10 code
CVD	I00-I25, I28-I99, E10.5, E11.5, E12.5, E13.5, E14.5
Ischaemic heart disease	I20-I25
Stroke	I60-I64
Other CVD	I00-I15, I28-I59, I65-I99, E10.5, E11.5, E12.5, E13.5, E14.5
Respiratory disease	I26-I27, J00-J11, J20-J99
COPD	I26-I27, J40-J44
Other respiratory disease	J00-J11, J20-J39, J45-J99
Chronic liver disease	B18-B19, B25.1, K70.0-K70.9, K72.1-K74, K75.2-K75.9, P35.3 Z22.5
Liver cirrhosis	K70.3, K74
Viral hepatitis	B18-B19, B25.1
Other liver disease	K70.0-K70.2, K70.4, K70.9, K72.1-K73, K75.2-K75.9, P35.3 Z22.5
Chronic kidney disease	I12.0-I13.9, E10.2, E11.2, E12.2, E13.2, E14.2, M10.3, M32.1, N02-N05, N08.3, N11-N13, N15, N18-N19, N25-N26, N27.1, N27.9, O10.2-O10.3, R94.4, T86.1, Z94.0
Infection	A00-B17, B20-B25, B25.2-B99, J12-J18
Pneumonia	J12-J18
Other infection	A00-B17, B20-B25.0, B25.2-B99
Cancer	C00-C97
Oesophageal	C15
Stomach	C16
Colorectal	C18-C20
Liver	C22
Pancreatic	C25
Lung	C33-C34
Female breast	C50
Female reproductive	C53, C54.1, C56
Other site	C00–C14, C17, C21, C23–C24, C26–C32, C35–C49, C51–C52, C54.0, C54.2-C55, C57-C97
External	V01-Y98
Other medical	Any ICD-10 code excluding the cause-specific mortality codes listed above

COPD=chronic obstructive pulmonary disease; CVD=cardiovascular disease

Table S2. Sensitivity analyses of the associations of usual random plasma glucose with mortality from selected major diseases, applying additional adjustments and/or exclusions

	All cause			Cardiovascular disease			Cancer			Infection			Chronic liver disease			Chronic kidney disease		
	n	HR*	95% CI	n	HR*	95% CI	n	HR*	95% CI	n	HR*	95% CI	n	HR*	95% CI	n	HR*	95% CI
Main model†	35700	1.14	(1.12-1.16)	13536	1.16	(1.12-1.16)	12316	1.12	(1.09-1.16)	686	1.19	(1.04-1.36)	522	1.45	(1.26-1.66)	369	1.44	(1.22-1.70)
Additionally adjusted for dietary variables‡	35700	1.14	(1.12-1.16)	13536	1.15	(1.12-1.19)	12316	1.12	(1.08-1.16)	686	1.19	(1.04-1.36)	522	1.44	(1.25-1.66)	369	1.44	(1.22-1.70)
Additionally adjusted for fasting time	35700	1.14	(1.12-1.16)	13536	1.15	(1.11-1.18)	12316	1.13	(1.09-1.17)	686	1.20	(1.04-1.37)	522	1.45	(1.25-1.67)	369	1.45	(1.22-1.72)
Additionally excluding the first 3 years of follow-up	29698	1.12	(1.10-1.14)	11572	1.15	(1.11-1.18)	10131	1.09	(1.05-1.13)	592	1.16	(1.01-1.34)	406	1.32	(1.12-1.56)	304	1.46	(1.21-1.75)
Additionally excluding participants who developed diabetes during follow-up	33912	1.14	(1.12-1.17)	12860	1.16	(1.13-1.19)	11814	1.13	(1.10-1.17)	646	1.21	(1.05-1.39)	499	1.48	(1.28-1.72)	311	1.34	(1.11-1.63)
Additionally excluding participants from Zhejiang	34139	1.14	(1.12-1.16)	13077	1.15	(1.12-1.19)	11438	1.12	(1.08-1.16)	666	1.20	(1.05-1.37)	505	1.43	(1.23-1.65)	356	1.50	(1.27-1.78)
Additionally excluding participants with poor self-rated health	30120	1.13	(1.11-1.16)	11257	1.14	(1.10-1.18)	10952	1.11	(1.07-1.15)	595	1.15	(1.00-1.33)	432	1.44	(1.23-1.68)	315	1.32	(1.09-1.59)

*HR per 1 mmol/L higher usual random plasma glucose

†Stratified by age-at-risk, sex and study area and adjusted for education, smoking, alcohol, physical activity, body mass index, waist and hip circumference and systolic blood pressure

‡Frequency of consumption of soybean products, preserved vegetables, fresh fruit, fresh vegetables, meat, poultry, fish/seafood, fresh eggs, dairy products, rice, wheat, other staple food

Vermunt J, *et al. BMJ Open Diab Res Care* 2021; 9:e002495. doi: 10.1136/bmjdrc-2021-002495

Table S3. Associations of baseline random plasma glucose with mortality from selected major diseases

Baseline RPG, mmol/L	All-cause			Cardiovascular disease			Cancer			Infection			Chronic liver disease			Chronic kidney disease		
	Deaths	HR	95% CI	Deaths	HR	95% CI	Deaths	HR	95% CI	Deaths	HR	95% CI	Deaths	HR	95% CI	Deaths	HR	(95% CI)
<4.8	5663	1.00	(0.97-1.03)	2031	1.00	(0.96-1.05)	1946	1.00	(0.96-1.05)	101	1.00	(0.81-1.22)	81	1.00	(0.80-1.25)	55	1.00	(0.76-1.31)
4.8 – 5.2	6353	1.01	(0.98-1.03)	2356	1.08	(1.03-1.12)	2242	0.99	(0.95-1.03)	123	0.97	(0.81-1.16)	86	1.01	(0.82-1.25)	48	0.82	(0.62-1.09)
5.3 – 5.7	6597	1.01	(0.99-1.03)	2432	1.06	(1.02-1.11)	2362	1.01	(0.97-1.05)	102	0.74	(0.61-0.90)	78	0.91	(0.72-1.13)	66	1.07	(0.84-1.36)
5.8 – 6.4	7304	1.06	(1.03-1.08)	2829	1.13	(1.09-1.17)	2543	1.06	(1.01-1.10)	139	0.96	(0.81-1.13)	110	1.21	(1.01-1.46)	79	1.20	(0.96-1.49)
6.5 – 7.5	5617	1.11	(1.08-1.14)	2179	1.14	(1.10-1.19)	1910	1.12	(1.07-1.17)	128	1.20	(1.01-1.43)	90	1.36	(1.10-1.67)	60	1.23	(0.95-1.58)
7.6 – 11.0	4166	1.28	(1.24-1.32)	1709	1.34	(1.27-1.40)	1313	1.22	(1.16-1.29)	93	1.35	(1.09-1.65)	77	1.83	(1.46-2.29)	61	1.99	(1.54-2.56)
≥11.1	1514	1.93	(1.84-2.04)	673	2.11	(1.95-2.28)	364	1.42	(1.28-1.57)	25	1.67	(1.12-2.48)	25	2.31	(1.55-3.45)	63	8.33	(6.42-10.8)
P for trend			*<0.001*			*<0.001*			*<0.001*			*0.002*			*<0.001*			*<0.001*
Per 1 mmol/L*	35700	1.14	(1.12-1.16)	13536	1.16	(1.12-1.19)	12316	1.12	(1.09-1.16)	686	1.19	(1.04-1.36)	522	1.45	(1.26-1.66)	369	1.44	(1.22-1.70)

*Excluding participants with baseline RPG <11.1 mmol/L

HRs are stratified by age-at-risk, sex and study area and adjusted for education, smoking, alcohol, physical activity, body mass index, waist and hip circumference and systolic blood pressure

RPG=random plasma glucose

Figure S1. Associations of usual random plasma glucose with mortality from selected major diseases

HRs are presented stratified by age-at-risk, sex and study area and adjusted for education, smoking, alcohol, physical activity, body mass index, waist and hip circumference and systolic blood pressure. HRs are plotted against mean usual RPG level in each category. Squares represent the HR with area inversely proportional to the variance of the log HR. Grey squares represent the HR among participants with screen-detected diabetes (baseline RPG ≥11.1 mmol/L). Vertical lines indicate the 95% CI. CVD=cardiovascular disease, RPG=random plasma glucose.

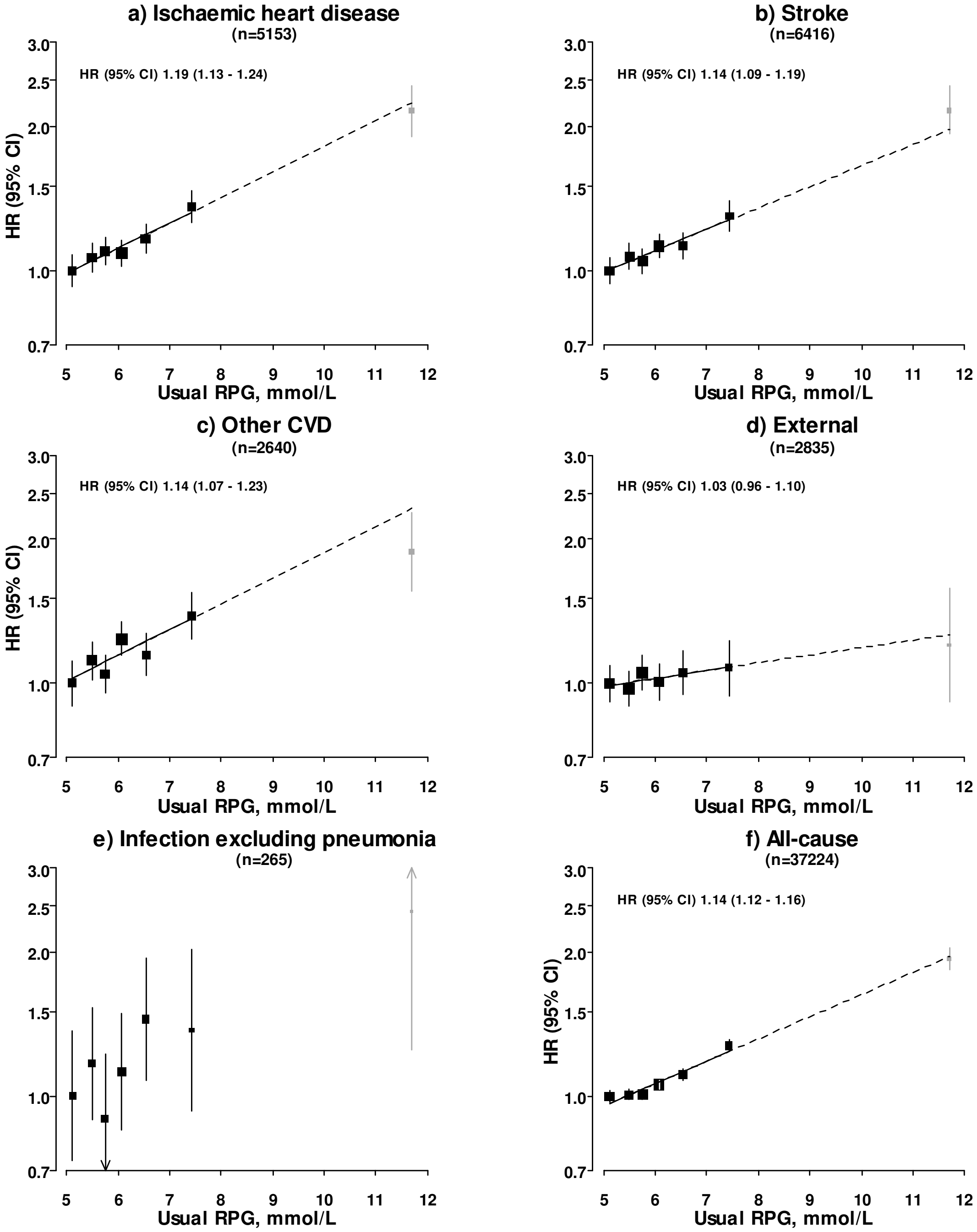

Vermunt J, *et al. BMJ Open Diab Res Care* 2021; 9:e002495. doi: 10.1136/bmjdrc-2021-002495

Figure S2. Associations of usual random plasma glucose with cancer mortality

HRs are presented stratified by age-at-risk, sex and study area and adjusted for education, smoking, alcohol, physical activity, body mass index, waist and hip circumference and systolic blood pressure. HRs are plotted against mean usual RPG level in each category. Squares represent the HR with area inversely proportional to the variance of the log HR. Grey squares represent the HR among participants with screen-detected diabetes (baseline RPG ≥11.1 mmol/L). Vertical lines indicate the 95% CI. RPG=random plasma glucose.

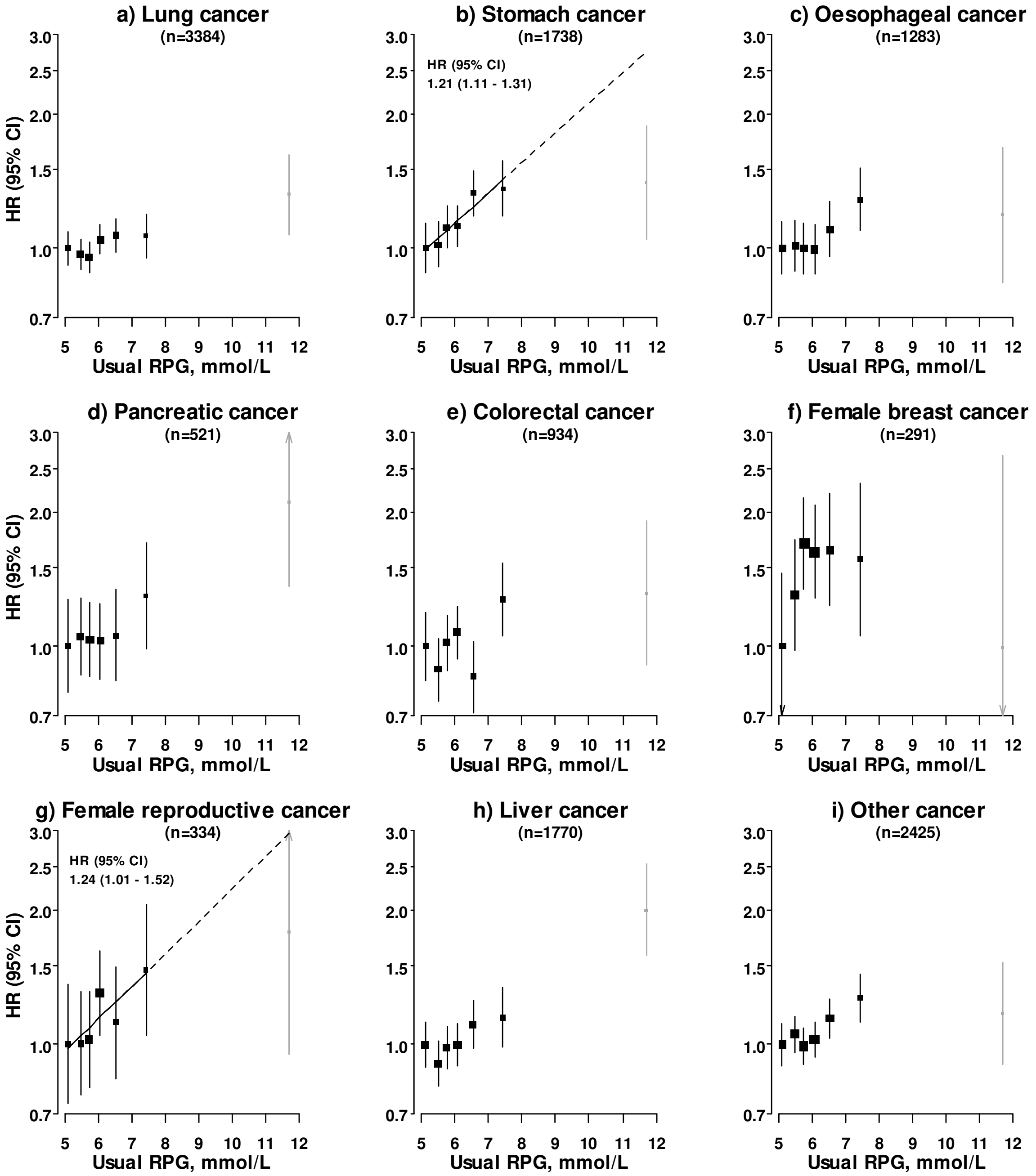

Vermunt J, *et al. BMJ Open Diab Res Care* 2021; 9:e002495. doi: 10.1136/bmjdrc-2021-002495

Figure S3. Associations of usual random plasma glucose with cardiovascular disease mortality

HRs are calculated per 1.0 mmol/L higher usual random plasma glucose. Results are stratified by age-at-risk, sex and study area and adjusted for education, smoking, alcohol, physical activity, body mass index, waist and hip circumference and systolic blood pressure, except where the variable of interest. Participants with screen-detected diabetes at baseline are excluded. Squares represent the HR with area inversely proportional to the variance of the log HR. Horizontal lines represent the corresponding 95% CI. The open diamond represents the overall HR and its 95% CI. BMI=body mass index; MET-h/day=metabolic equivalent of task hours per day; SBP=systolic blood pressure.

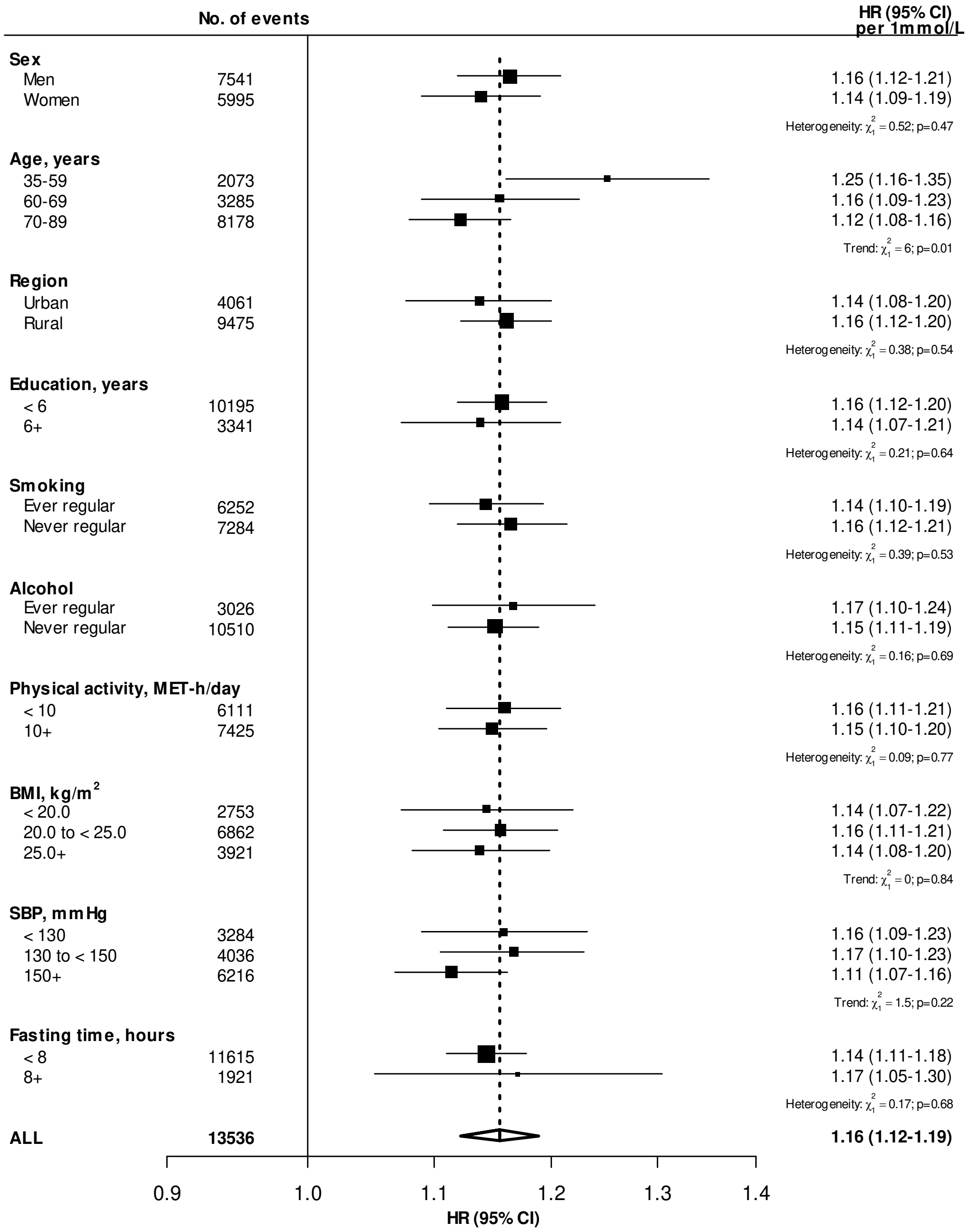

Figure S4. Associations of usual random plasma glucose with all-cause mortality in selected population subgroups

HRs are calculated per 1.0 mmol/L higher usual random plasma glucose. Results are stratified by age-at-risk, sex and study area and adjusted for education, smoking, alcohol, physical activity, body mass index, waist and hip circumference and systolic blood pressure, except where the variable of interest. Participants with screen-detected diabetes at baseline are excluded. Squares represent the HR with area inversely proportional to the variance of the log HR. Horizontal lines represent the corresponding 95% CI. The open diamond represents the overall HR and its 95% CI. BMI=body mass index; MET-h/day=metabolic equivalent of task hours per day; RPG=random plasma glucose; SBP=systolic blood pressure.

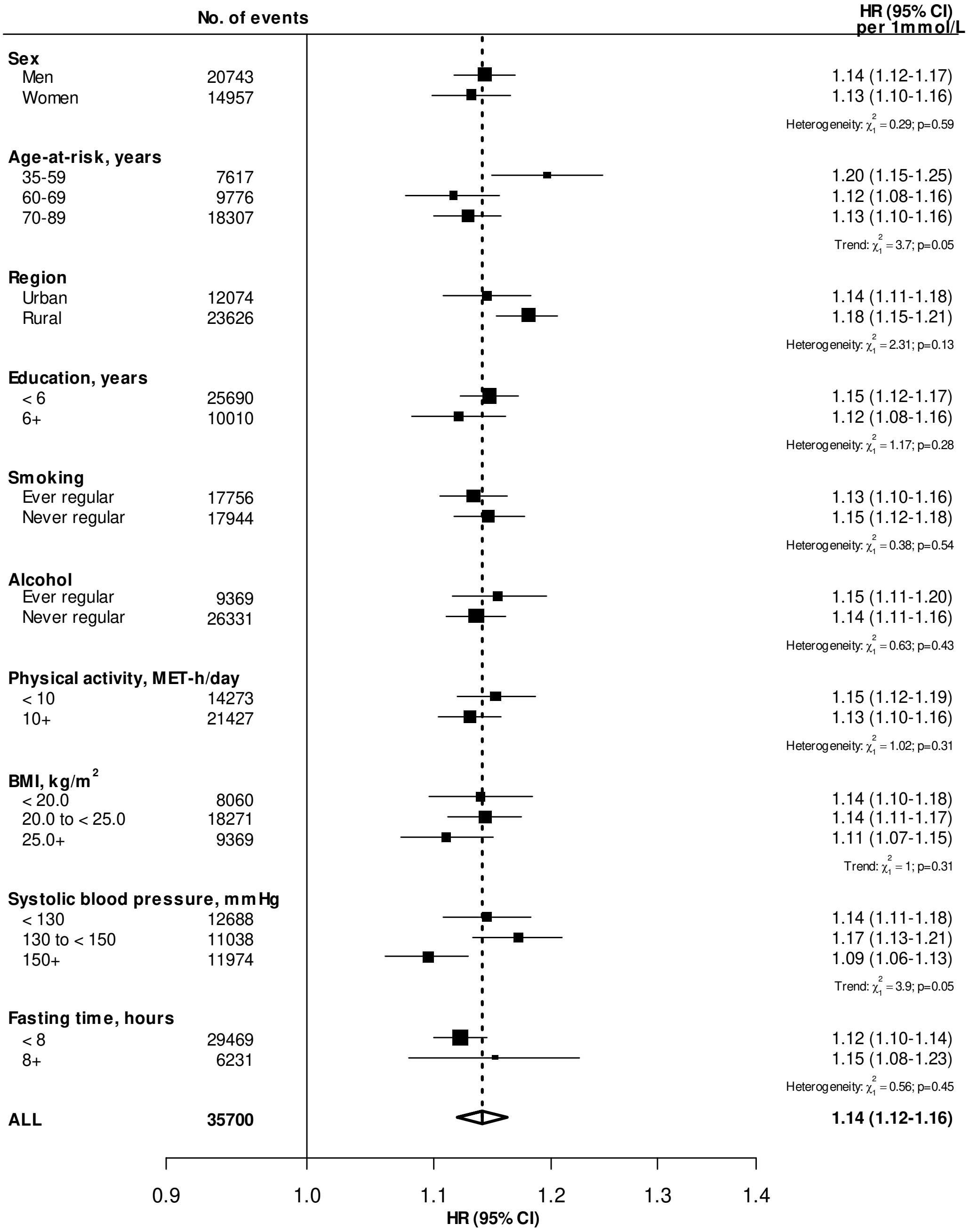

Figure S5. Associations of usual random plasma glucose with cancer mortality

HRs are calculated per 1.0 mmol/L higher usual random plasma glucose. Results are stratified by age-at-risk, sex and study area and adjusted for education, smoking, alcohol, physical activity, body mass index, waist and hip circumference and systolic blood pressure, except where the variable of interest. Participants with screen-detected diabetes at baseline are excluded. Squares represent the HR with area inversely proportional to the variance of the log HR. Horizontal lines represent the corresponding 95% CI. The open diamond represents the overall HR and its 95% CI. BMI=body mass index; MET-h/day=metabolic equivalent of task hours per day; SBP=systolic blood pressure.

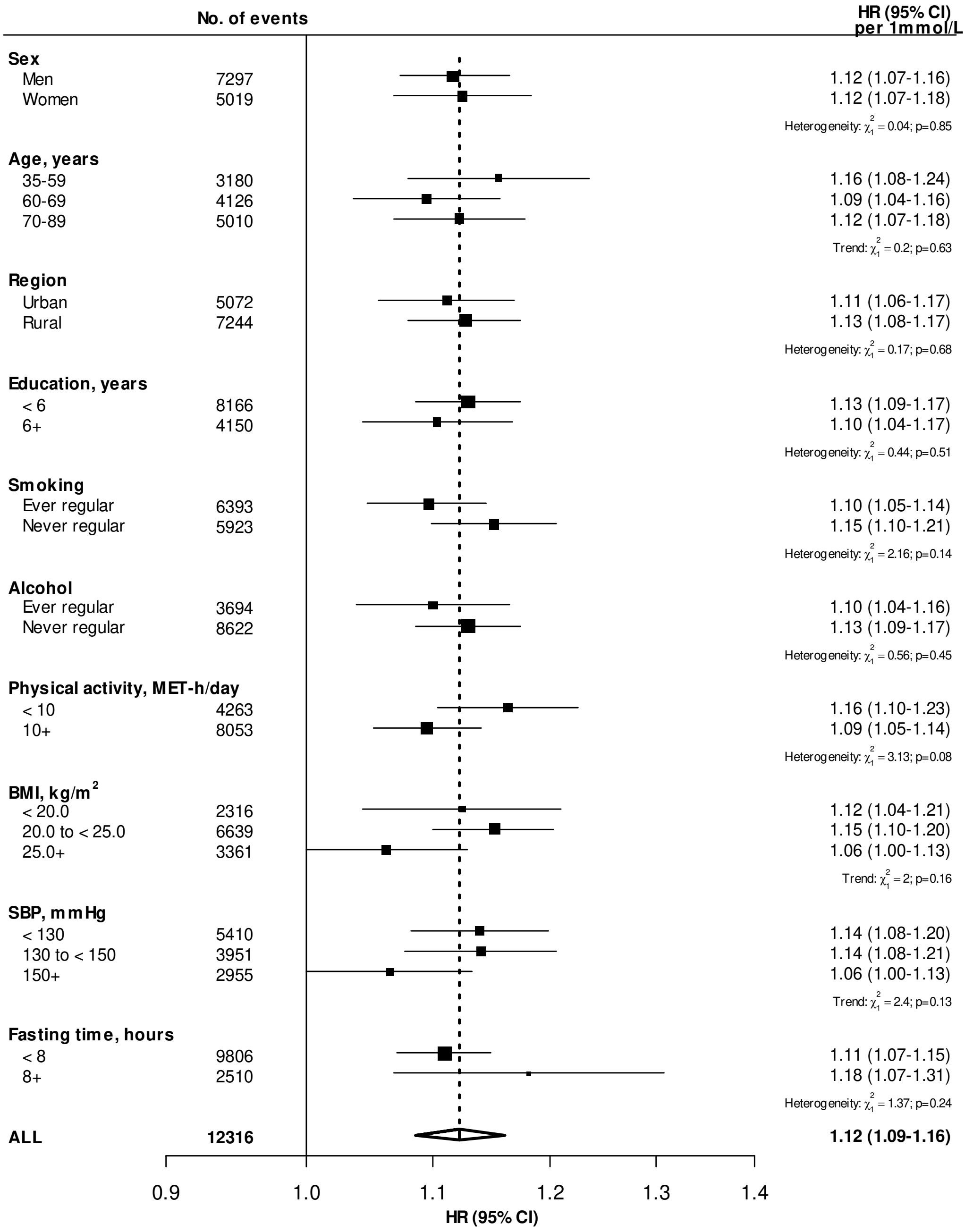

Figure S6. Associations of usual random plasma glucose with all-cause, cardiovascular disease and cancer mortality by study area

HRs are calculated per 1.0 mmol/L higher usual random plasma glucose. Results are stratified by age-at-risk and sex and adjusted for education, smoking, alcohol, physical activity, body mass index, waist and hip circumference and systolic blood pressure. Participants with screen-detected diabetes at baseline are excluded. Squares represent the HR with area inversely proportional to the variance of the log HR. Horizontal lines represent the corresponding 95% CI. The open diamond represents the overall HR and its 95% CI.

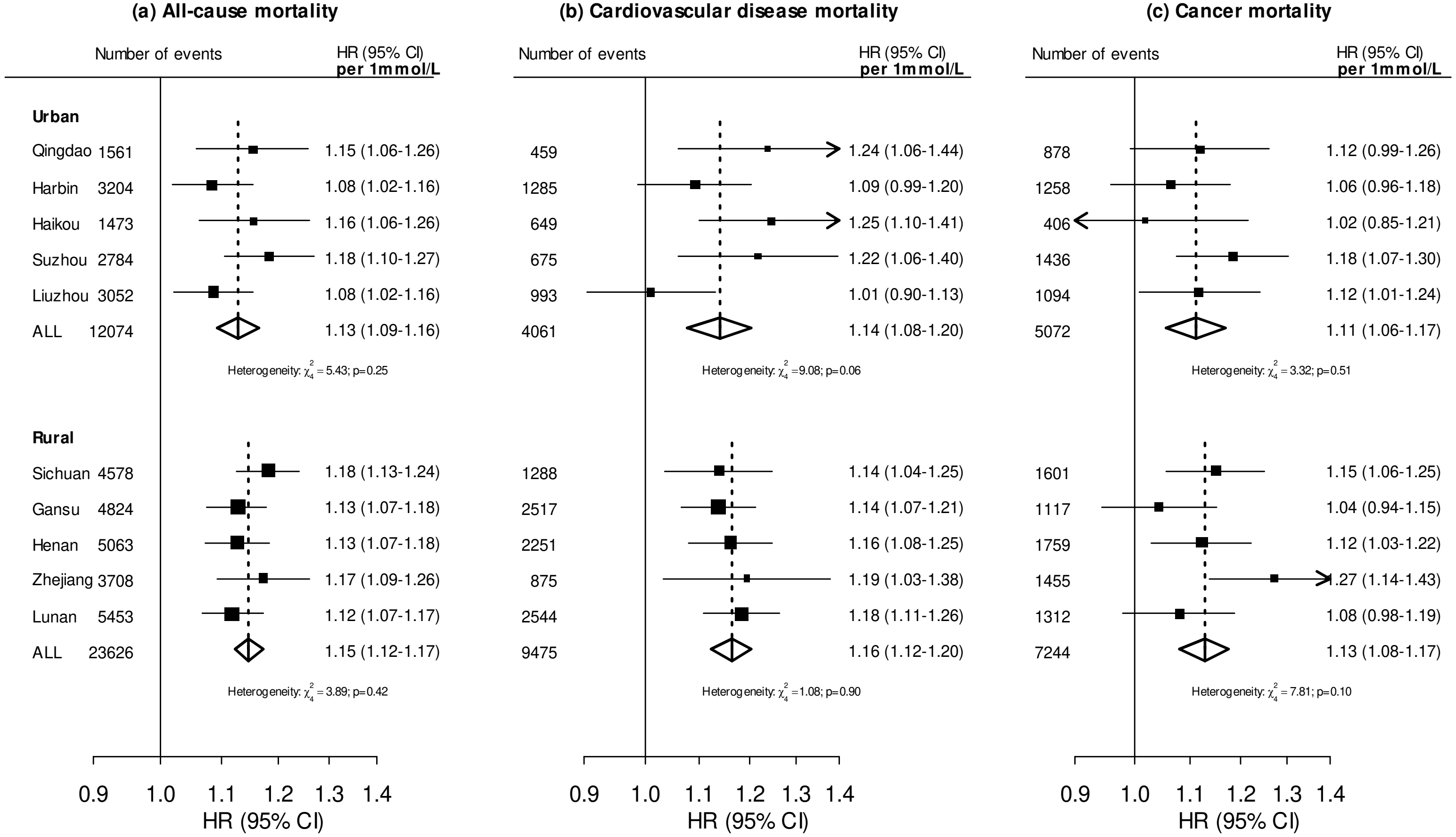

Vermunt J, et al. BMJ Open Diab Res Care 2021; 9:e002495. doi: 10.1136/bmjdrc-2021-002495

Figure S7. Associations of usual random plasma glucose with liver disease mortality

HRs are presented stratified by age-at-risk, sex and study area and adjusted for education, smoking, alcohol, physical activity, body mass index, waist and hip circumference and systolic blood pressure. HRs are plotted against mean usual RPG level in each category. Squares represent the HR with area inversely proportional to the variance of the log HR. Grey squares represent the HR among participants with screen-detected diabetes (baseline RPG ≥11.1 mmol/L). Vertical lines indicate the 95% CI. RPG=random plasma glucose.

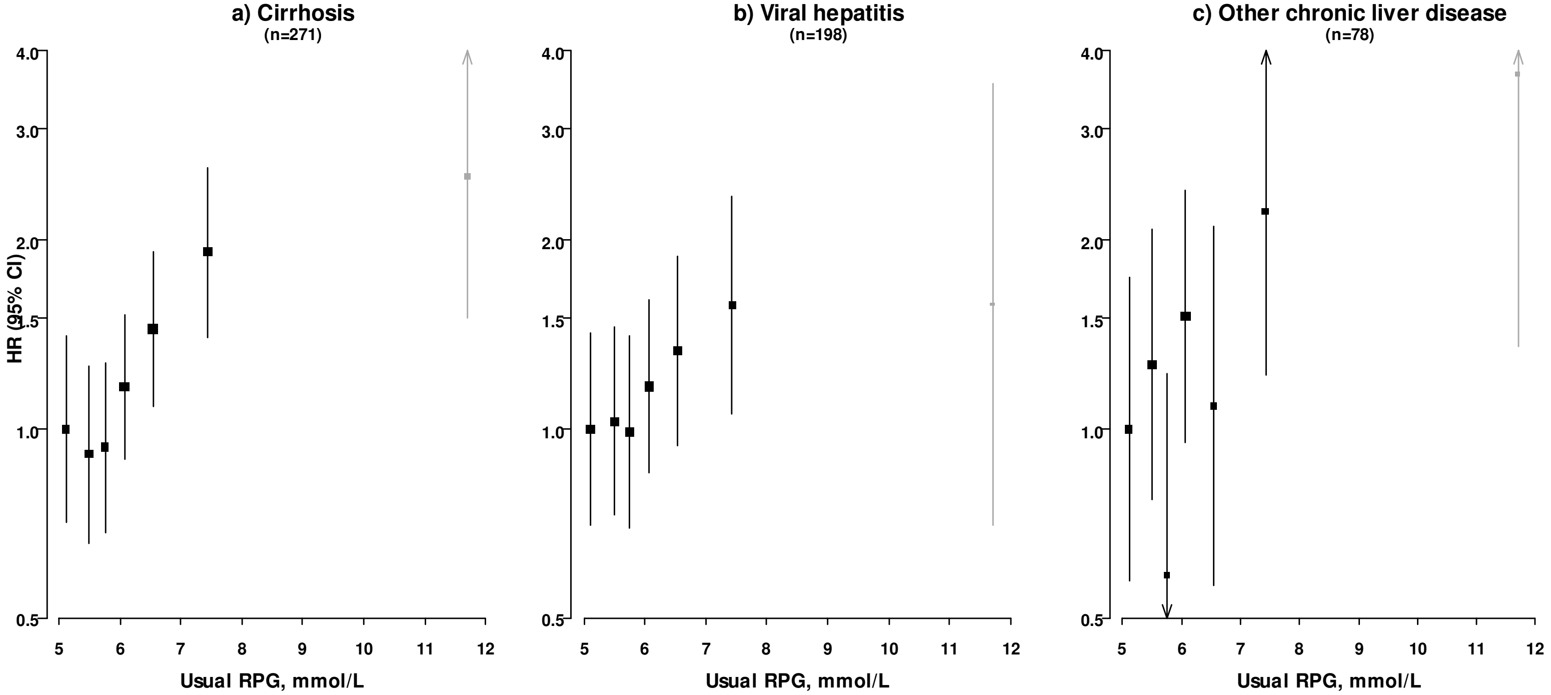

Figure S8. Associations of usual random plasma glucose with respiratory disease mortality

HRs are presented stratified by age-at-risk, sex and study area and adjusted for education, smoking, alcohol, physical activity, body mass index, waist and hip circumference and systolic blood pressure. HRs are plotted against mean usual RPG level in each category. Squares represent the HR with area inversely proportional to the variance of the log HR. Vertical lines indicate the 95% CI. Grey squares represent the HR among participants with screen-detected diabetes (baseline RPG ≥11.1 mmol/L). COPD=chronic obstructive pulmonary disease; RPG=random plasma glucose.

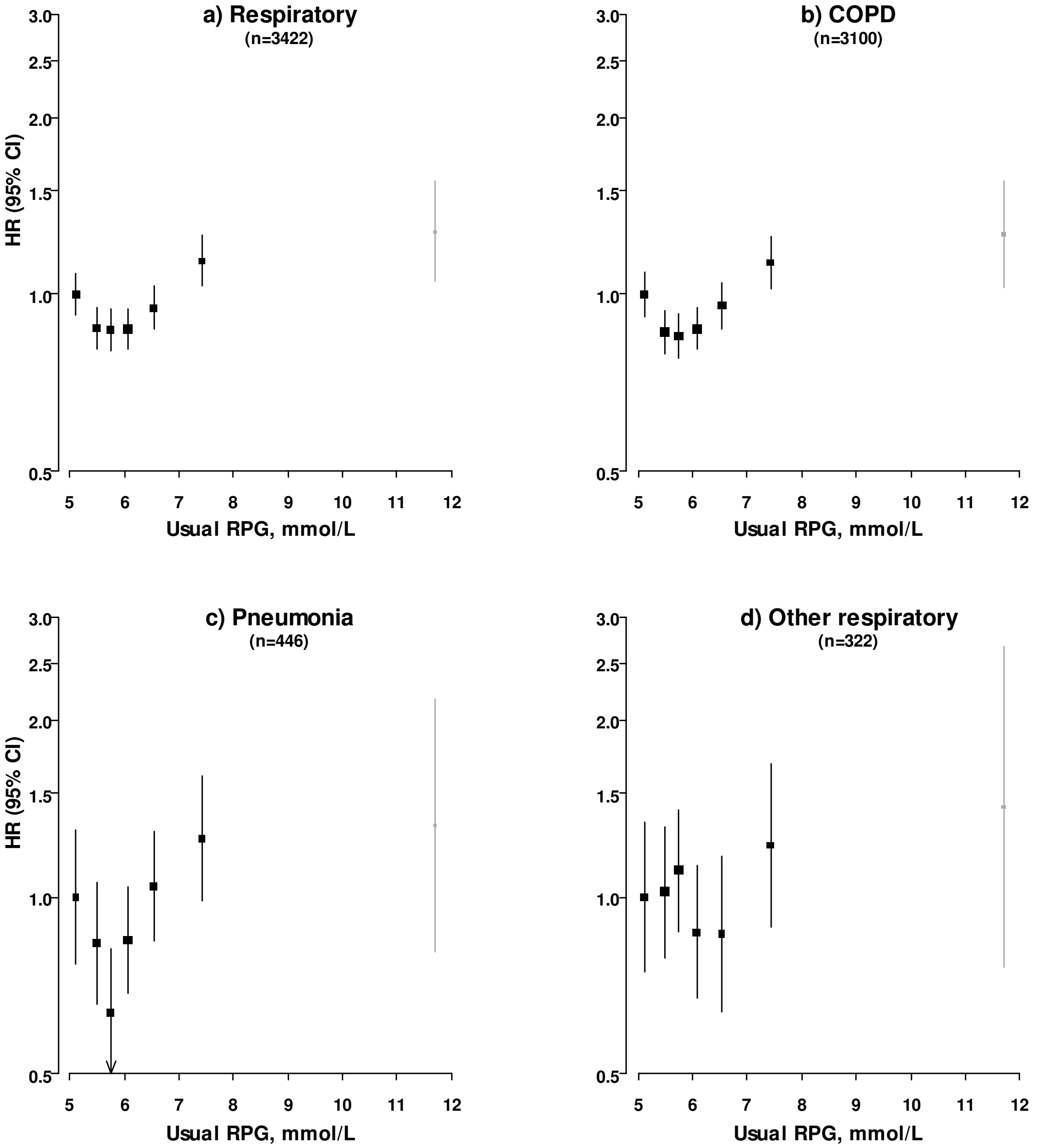

Vermunt J, *et al. BMJ Open Diab Res Care* 2021; 9:e002495. doi: 10.1136/bmjdrc-2021-002495
